# Identification of antigens in the *Trichinella spiralis* extracellular vesicles for serological detection of early stage infection in swine

**DOI:** 10.1186/s13071-023-06013-7

**Published:** 2023-10-26

**Authors:** Chengyao Li, Chen Li, Fengyan Xu, Haolu Wang, Xuemin Jin, Yuanyuan Zhang, Xiaolei Liu, Ruizhe Wang, Xihuo You, Mingyuan Liu, Xue Bai, Yong Yang

**Affiliations:** 1 State Key Laboratory for Diagnosis and Treatment of Severe Zoonotic Infectious Diseases, Key Laboratory for Zoonosis Research of Ministry of Education, Institute of Zoonosis, and College of Veterinary Medicine, Jilin University, Changchun, China; 2 School of Basic Medical Science, Shan Xi Medical University, Taiyuan, China; 3 Jiangsu Co-innovation Center for Prevention and Control of Important Animal Infectious Diseases and Zoonoses, Yangzhou, Jiangsu People’s Republic of China; 4 Beijing Agrichina Pharmaceutical Co., Ltd, Wangzhuang Industrial Park, Airport Road, Shahe, Changping District, Beijing, China

**Keywords:** *Trichinella spiralis*, Detection antigens of early stage, Proteomics, Extracellular vesicle

## Abstract

**Background:**

Several studies have reported the roles of *Trichinella spiralis* extracellular vesicles in immune regulation and pathogen diagnosis. Currently, the *T. spiralis* muscle larvae excretory/secretory product (*Ts*-ML-ES) is the antigen recommended by the International Commission on Trichinellosis (ICT) for serological diagnosis of trichinellosis. However, it can only be used to detect middle and late stages of infections, and cross-reactions with other parasite detections occur. Therefore, there is a need to identify antigens for specific detection of early stage trichinellosis.

**Methods:**

Extracellular vesicles of *T. spiralis* muscle larvae (*Ts*-ML-EVs) were isolated by ultracentrifugation and characterized by transmission electron microscopy, nanoparticle tracking analysis, flow cytometry and western blot. *Ts*-ML-EVs protein profiles were analyzed by LC-MS/MS proteomics for identification of potential antigens (*Ts*-TTPA). *Ts*-TTPA were cloned into pMAL-c5X vector and expressed as recombinant proteins for evaluation of potential as detected antigens by western blot and ELISA.

**Results:**

Isolated *Ts*-ML-EVs were round or elliptic (with diameters between 110.1 and 307.6 nm), showing a bilayer membrane structure. The specific surface markers on the *Ts*-ML-EVs were CD81, CD63, enolase and the 14-3-3 protein. A total of 53 proteins were identified by LC-MS/MS, including a variety of molecules that have been reported as potential detection and vaccine candidates. The cDNA of *Ts*-TTPA selected in this study has a total length of 1152 bp, encoding 384 amino acids with a molecular weight of 44.19 kDa. It contains a trypsin domain and can be recognized by anti-His antibody. It reacted with swine sera infected with 10,000 *T. spiralis* at 15, 25, 35 and 60 days post-infection (dpi). At 10 μg/ml, this antigen could detect *T. spiralis* antibodies from the swine sera at 13 dpi. There were no cross-reactions with the swine sera infected with other parasites including *Clonorchis sinensis*, *Toxoplasma gondii*, *Taenia suis*, *Ascaris suis* and *Trichuris suis*.

**Conclusions:**

This study identifies potential early stage detection antigens and more thoroughly characterizes a serine protease domain-containing protein. Extracellular vesicle proteins may be explored as effective antigens for the early stage detection of trichinellosis.

**Graphical Abstract:**

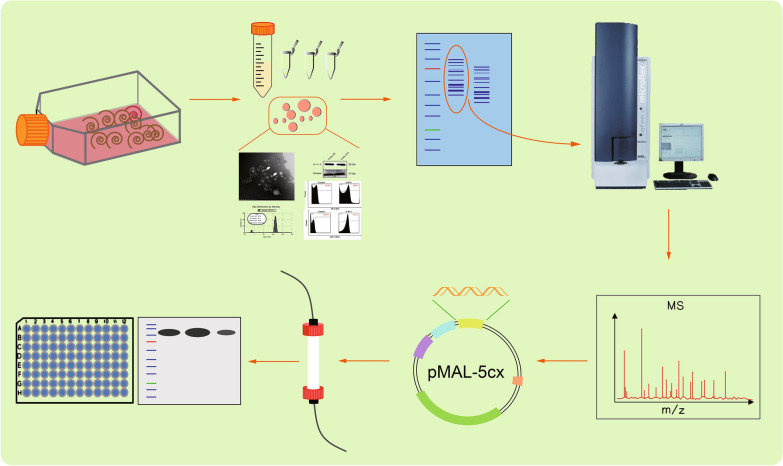

## Backgrounds

*Trichinella spiralis* is an important foodborne parasite that causes trichinellosis in a wide variety of hosts, causing significant public health and economic impacts. Hosts are usually infected by ingestion of raw or undercooked meats containing the cysts of muscle larvae [1]. After digestion in the stomach, the cysts develop into intestinal infective larvae and undergo four molts to grow into adults in the intestine. The adult worms travel through the intestinal epithelium where they mate and produce offspring. The newborn larvae pass through the host circulatory system to reach the muscle cells for permanent parasitism [2, 3].

The most commonly used *Ts*-ML-ES antigen is recommended by the International Commission on Trichinellosis (ICT) for serological detection for trichinellosis. However, previous studies have shown that 100% detection of anti-*T. spiralis* IgG antibodies by ES-ELISA are unlikely for at least 1–3 months after the primary *T. spiralis* infection [4]. There is a clear window of 3–4 weeks between infection and specific antibody positivity [5]. Therefore, there is a need to identify antigens for detecting early stage *T. spiralis* infection. Some studies have reported screening of early stage antigens from adult worm crude extracts and excretory-secretory products [6]. A number of early antigens have been identified from cDNA subtractive libraries and proteomics, but they generally lack specificity and sensitivity [7, 8].

Extracellular vesicles (EVs) are vesicles secreted by a variety of cells and can be classified into micro-vesicles, exosomes, apoptotic bodies and oncosomes [9]. EVs may participate in parasite-host interactions and host immune regulation. EV proteins are also explored for detection and diagnosis, including those from the parasites [10–13]. For example, Kifle et al. identified 15,120-kDa EV proteins from *Schistosoma mansoni* and showed their potential to serve as vaccine candidates for schistosomiasis [14]. Our previous study also showed that *T. spiralis* muscle larval EVs (Ts-ML-EVs) contained fewer proteins than the muscle larval excretory-secretory products (*Ts*-ML-ES) but produced stronger host immunological responses [15].

In this study, we used LC-MS/MS technology combined with bioinformatics analysis to analyze EV proteins from *T. spiralis* muscle larvae, aiming to identify antigens that could detect antibodies from the swine sera at the early stage of *T. spiralis* infection. We found that *Ts*-TTPA protein with trypsin domain identified swine sera at 13 dpi most quickly and is a candidate antigen for early stage detection of trichinellosis.

## Methods

### Animals and *T. spiralis*-infected swine sera

Female Wistar rats (~ 200 g) were purchased from the Experimental Animal Center of Jilin University.

The swine sera used in the experiment included 7, 9, 11, 13, 15, 17, 19, 21, 25, 30, 35, 45, 60, 90 and 120 dpi of swine serum infected with 10,000 *T. spiralis*. The serum of pigs not infected with *T. spiralis* was used as negative control. The above swine serum is from our team's previous research [5]. Similarly, the swine serum used in the cross-reactions was derived from previous studies in the laboratory [16].

### Parasite maintenance and collection

The *T. spiralis* (ISS 534 strain) used in this study was maintained in female Wistar rats in our laboratory (3500 ML/rat, maintained for about 6 weeks after oral infection), and *T. spiralis* muscle larvae were recovered from the muscles of infected rats with a standard pepsin/hydrochloric acid digestion method [17]. After washing with normal saline containing 2 × penicillin-streptomycin (Biological Industries, Kibbutz Beit Haemek, Israel), 5000 muscle larvae/ml were cultured in RIPM1640 medium (Gibco, California, USA) containing 2 × penicillin-streptomycin at a 37 °C for 18 h [5].

### Isolation of extracellular vesicles of* T. spiralis* muscle larvae (*Ts-*ML-EVs)

The above cultures were collected in 50-ml tubes and centrifuged at 800 g for 10 min followed by 5000 g for 20 min to remove excess worm fragments and other large impurities. The supernatant was then passed through a 0.22-μm filter (Millipore, Darmstadt, Germany) to remove larger vesicles and bacteria. Centrifugation was performed using a 10-kDa ultrafiltration tube (Millipore, Darmstadt, Germany) at 5000 g to concentrate the liquid to the appropriate volume. The supernatant was then ultracentrifuged at 4 °C, 120,000 g for 2 h with an ultracentrifuge (Hitachi, Tokyo, Japan). After the supernatant was discarded carefully, sterile PBS (phosphate buffer saline, PH = 7.4) was used to suspend the bottom vesicles, and the protein concentration of the collected *Ts*-ML-EVs was measured with a bicinchoninic acid (BCA) protein assay kit (Beyotime, Beijing, China). Samples were individually packaged in tubes and stored at − 80 °C for the next steps.

### Characterization of *Ts*-ML-EVs by transmission electron microscopy, nanosight, western blot and flow cytometry

Negative staining transmission electron microscopy (TEM) was used to analyze the morphology, structure and size of the *Ts*-ML-EVs. Briefly, 10 μl of the *Ts*-ML-EVs suspension was dropped on the copper mesh, adsorbed for 1 min and then soaked in 2% glutaraldehyde at room temperature. The grids were then negatively stained with 2% phosphotungstic acid for 1 min and air-dried at room temperature. The *Ts*-ML-EVs were examined at 80 kV using a Hitachi H-7650 transmission electron microscope (Hitachi, Tokyo, Japan). The *Ts*-ML-EVs particles were characterized for size and concentration using a NanoSight NS300 (Malvern Panalytical, UK). Then, western blotting (WB) and flow cytometry were used for surface marker identification. For WB, 15 μg *Ts*-ML-EVs and 15 μg *Ts*-ML-ES) were electrophoresed in 12% SDS-PAGE (sodium dodecyl sulfate-polyacrylamide gel electrophoresis) and transferred to PVDF membranes (Immobilon, Millipore, USA). The membranes were blocked with 5% skim milk and incubated with primary antibodies including polyclonal rabbit anti-CD63 (1:1000, Abcam, Cambridge, UK) and polyclonal goat anti-enolase (1:200, Abcam, Cambridge, UK). Subsequently, two different horseradish peroxidase (HRP)-conjugated secondary antibodies were used: goat anti-rabbit IgG (1:2000, Cell Signaling Technology, USA) and donkey anti-goat IgG (1:50,000, Jackson ImmunoResearch, USA). The membrane was visualized using an ECL luminescence chromogenic solution (Thermofisher Scientific, Waltham, MA, USA) and a chemiluminescence instrument (Analytik Jena, Thuringia, Germany). For flow cytometry, the *Ts*-ML-EVs were diluted to 100 μg/ml in 100 μl PBS, and PE-Mouse Anti-Human CD63 (Clone: H5C6, BD Biosciences, New Jersey, USA) and APC-Mouse Anti-Human CD81 (Clone: JS-81, BD Biosciences, NJ, USA) antibodies were added. The samples were incubated at room temperature for 30 min in the dark and then ultracentrifuged for 15 min at 4 °C, 120,000 g. The supernatant was discarded, and PBS was added to resuspend the bottom vesicles. Finally, a flow cytometer was used (BD FACS Aria, BD Biosciences, NJ, USA) for vesicle detection.

### SDS-PAGE and western blot analysis

The SDS-PAGE analyses were performed on the *Ts*-ML-ES (15 μg) and *Ts*-ML-EVs (15 μg) on three gels, respectively. One of these was transferred to a membrane for blocking, and the bands were displayed by hybridization using swine serum (1:200) of uninfected or 25 dpi with 10,000 *T. spiralis* muscle larvae. One of the gels was stained with Coomassie Bright Blue Fast Staining solution (P1300, Solarbio, Beijing, China) and destained with destaining solution. Then, a band at the same position as the hybridized band was cut out and sequenced. Another gel was used for silver staining. Specific protocols refer to the manual provided by the manufacturer (P0017S, Beyotime, Beijing, China).

The recombinant protein (r*Ts*-TTPA) was performed on 12% SDS-PAGE. After protein transfer and blocking, swine sera at 15, 25, 35, 60 and 120 dpi with 10,000 *T. spiralis* and negative serum (1:200) or mouse His-tag antibody (1:1000) were used as primary antibodies, while HRP-goat anti-pig IgG (Abcam, Massachusetts, UK) and HRP-goat anti-mouse IgG were used as secondary antibodies (1:2000). Chemiluminescence substrates were used to detect protein bands through UVP ChemStudio (Analytik Jena, Thuringia, Germany).

### In-gel digestion

For in-gel tryptic digestion, gel pieces were destained in 50 mM NH_4_HCO_3_ in 50% acetonitrile (v/v) until clear. Gel pieces were dehydrated with 100 μl of 100% acetonitrile for 5 min, the liquid removed and the gel pieces rehydrated in 10 mM dithiothreitol before being incubated at 56 °C for 60 min. Gel pieces were again dehydrated in 100% acetonitrile. The liquid was removed and gel pieces rehydrated with 55 mM iodoacetamide. Samples were then incubated at room temperature, in the dark, for 45 min. Gel pieces were washed with 50 mM NH_4_HCO_3_ and dehydrated with 100% acetonitrile. Gel pieces were rehydrated with 10 ng/μl trypsin, and resuspended in 50 mM NH_4_HCO_3_ on ice for 1 h. Excess liquid was removed, and the gel pieces were digested with trypsin at 37 °C overnight. Peptides were extracted with 50% acetonitrile/5% formic acid, followed by 100% acetonitrile. Peptides were dried to completion and resuspended in 2% acetonitrile/0.1% formic acid for further analysis.

### LC-MS/MS analysis

The tryptic peptides were dissolved in 0.1% formic acid (Fluka, Seelze, Germany) (solvent A) and directly loaded onto a homemade reversed-phase analytical column (15 cm long, 75 μm i.d.). The gradient was comprised of an increase from 6 to 23% solvent B [0.1% formic acid in 98% acetonitrile (Fisher Chemical, Waltham, USA)] and ran over 16 min, 23% to 35% over 8 min, finally climbing to 80% over 3 min and then holding at 80% for the last 3 min. This was performed at a constant flow rate of 400 nl/min on an EASY-nLC 1000 UPLC system (Thermo Fisher Scientific, Waltham, MA, USA).

The peptides were subjected to an NSI source followed by tandem mass spectrometry (MS/MS) in a Q ExactiveTM Plus (Thermo Fisher Scientific, Waltham, MA, USA) coupled online to the UPLC. The electrospray voltage applied was 2.0 kV. The m/z scan range was 350–1800 for a full scan, and intact peptides were detected in the Orbitrap (Thermo Fisher Scientific, Waltham, USA) at a resolution of 70,000. Peptides were then selected for MS/MS using an NCE setting of 28, and the fragments were detected in the Orbitrap at a resolution of 17,500. This data-dependent procedure alternated between one MS scan followed by 20 MS/MS scans with 15.0 s dynamic exclusion. Automatic gain control (AGC) was set at 5E4.

### Data processing

The resulting MS/MS data were processed using Proteome Discoverer 2.4. Tandem mass spectra were searched blasted against the *T. spiralis* database (18,572) sequences. Trypsin/P was specified as a cleavage enzyme allowing up to two missing cleavages. A mass error was set to 10 ppm for precursor ions and 0.02 Da for fragment ions. Carbamidomethyl on Cys was specified as a fixed modification, and oxidation was specified as a variable modification on Met. Peptide confidence was set at high, and peptide ion score was set at > 20.

### ELISA

Ten μg/ml r*Ts*-TTPA in 100 μl CBS (carbonate buffer solution) was coated on an EIA-ELISA plate at 4 °C overnight. After the encapsulation solution was discarded, it was blocked with 200 μl 5% skim milk at 37 °C for 1 h, and then PBST (phosphate buffer saline with 2% Tween-20) was used for three 1-min washes. After patting the liquid dry, serum collected from pigs on different days post-infection with 10,000 *T. spiralis* or serum infected with *Clonorchis sinensis*, *Toxoplasma gondii*, *Taenia suis*, *Ascaris suis* and *Trichuris suis*, diluted with a blocking solution (1:100), was added for the binding reaction. After the same washing and patting, HRP-goat-anti-pig secondary antibodies (Abcam, Cambridge, UK) in a blocking solution (1:5000) were incubated at 37 °C for 45 min. After washing and termination, TMB substrate (Tiangen, Beijing, China) was added to detect absorbance at 450 nm (Biotek, Vermont, USA).

### Protein expression and purification

The protein (named tissue-type plasminogen activator, *Ts*-TTPA) was identified after LC-MS/MS analysis. The expression vector pMAL-c5X was cloned with BamHI-EcoRI, and these were transformed into BL21 (DE3) for protein expression. By optimizing the conditions, the soluble r*Ts*-TTPA was obtained. The induction conditions were 1 M IPTG at 16 °C for 20 h. The purified protein was obtained by repeated freeze-thawing, ultrasonic crushing and Ni-affinity chromatography (Qiagen, Dusseldorf, Germany) according to the manufacturer’s instructions.

### Bioinformatics analysis

Secondary structure prediction of proteins was performed with Protein Structure Prediction Server [18]. Domain analysis was performed with Pfam [19]. A Swiss model was used to build the 3D protein structure [20]. Isoelectric point prediction was completed in ProtParam [21]. The GO analysis was completed in Gene Ontology Resource [22]. Molecular Evolutionary Genetics Analysis software (MEGA10) [23] was used to draw the maximum likelihood tree.

### Statistical analysis

GraphPad Prism 9 (9.0.0) was used for statistical analysis and visualization with one-way ANOVA followed by Tukey’s multiple comparison post-test.

## Results

### Characterization of *Ts*-ML-EVs

**Fig. 1:**
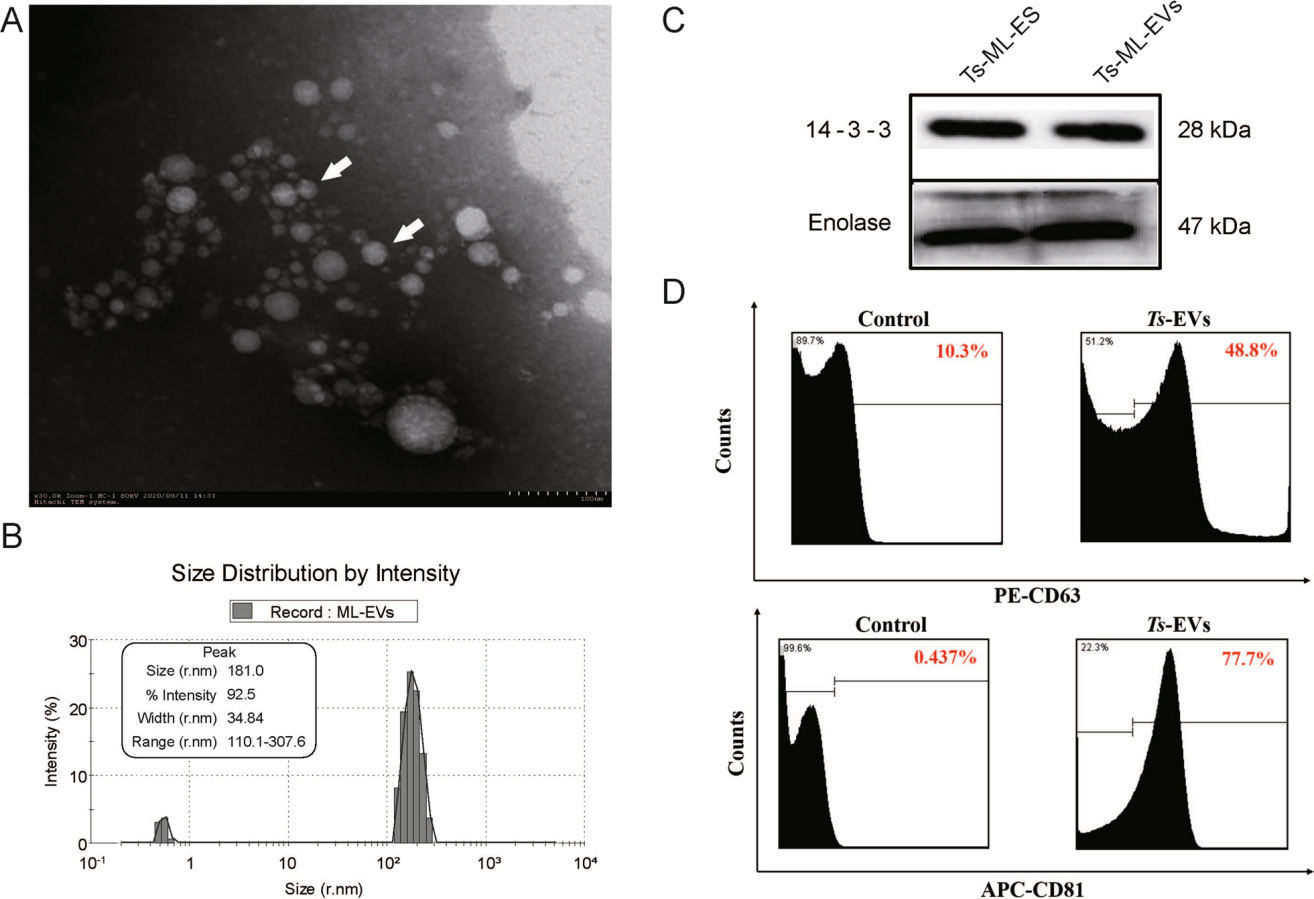
Characterization and identification of *Ts*-ML-EVs. **A** The morphology and size of *T. spiralis* extracellular vesicles visualized by scanning electron microscopy. White arrow indicates the extracellular vesicles (scale bar 100 μM). **B** NTA shows the size distribution of *T. spiralis* extracellular vesicle by intensity. The illustration is a combination of histograms and peak charts. **C** Identification of marker proteins 14-3-3, enolase in the excretory-secretory product (40 μg) and extracellular vesicles (40 μg) of *T. spiralis*. **D** CD81 and CD63 were identified on the extracellular vesicles of *T. spiralis* by flow cytometry

The *Ts*-ML-EVs were characterized as typical circular or oval bilayer membrane-like vesicles with different sizes (Fig. 1a), ranging from 110.1–307.6 nm, and peak sizes were 181 nm (Fig. 1b). We confirmed by western blot, the *Ts*-ML-EVs contained the EV marker proteins Enolase and 14-3-3 (Fig. 1c). In addition, flow cytometry showed that CD63 and CD81 were the specific markers on the surface of the *Ts*-ML-EVs, and the positive rates were 48.8% and 77.7%, respectively (Fig. 1d). These results suggest that the extracellular vesicles collected in this study conformed to the expected characteristics of extracellular vesicles and can be used in further experiments.

### *Ts*-ML-EVs can be recognized by swine serum at 25 days post-infection

**Fig. 2:**
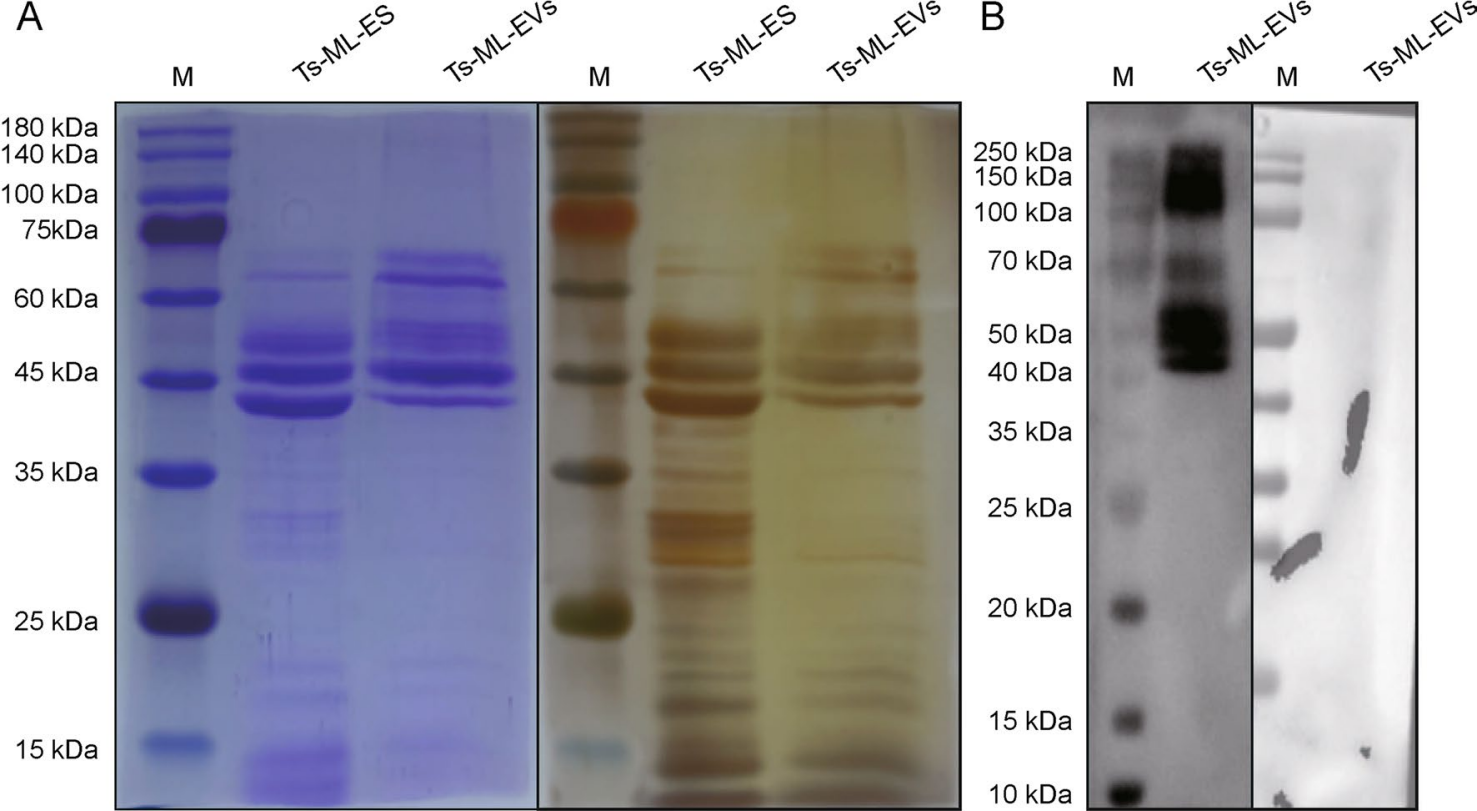
Determination of components of Ts-ML-ES and Ts-ML-EVs. **A** SDS-PAGE of *Ts*-ML-ES and *Ts*-ML-EVs stained with Coomassie Bright Blue and silver. **B** *Ts*-ML-ES and *Ts*-ML-EVs were identified by serum at 25 dpi with 10,000 *T. spiralis*. Negative serum was used as a control

Both Coomassie Blue and silver stainings showed that *Ts*-ML-EVs had many bands, with molecular weights ranging from 10 to 180 kDa (Fig. 2a). Western blot analysis showed that the bands of *Ts*-ML-EVs that could be reacted with 25 dpi swine serum were concentrated between 40 and 180 kDa. However, negative swine serum did not recognize these protein bands (Fig. 2b).

### Protein identification by LC-MS/MS analysis

**Fig. 3:**
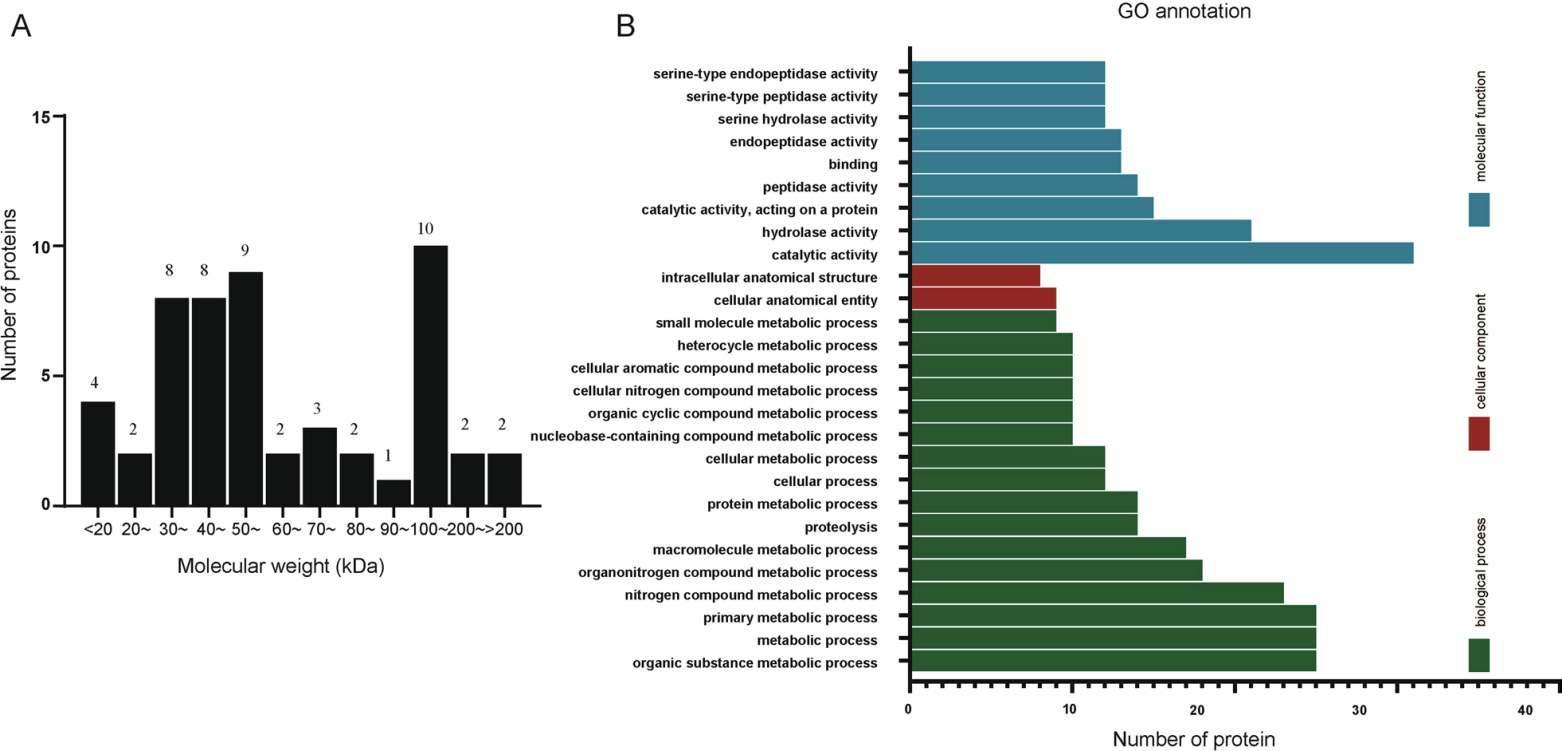
Molecular weight and GO annotation of proteins screened by LC-MS/MS. **A** The distribution of all 53 protein numbers with different molecular weights was detected by LC-MS/MS. **B** The GO annotation shows the number of proteins involved in different functions detected by LC-MS/MS

The protein bands identified by 25 dpi with 10,000 *T. spiralis* early infection sera were separately analyzed by LC-MS/MS, and a total of 200 unique *T. spiralis* extracellular vesicle proteins with a pep count ≥ 2 were identified by searching the *T. spiralis* database in UniProt. Except for the redundant sequences, they were clustered into 53 unique proteins. The molecular weight (MW) of 53 proteins ranged from 10 to 484 kDa, of which 35 (66.03%) proteins were distributed in the range of 30–60 kDa (Table 1, Fig. 3a). The enrichment analysis of these proteins shows that these proteins have cellular components, molecular functions and biological processes (Fig. 3b). There are 25 proteins involved in the organic substance metabolic process, metabolic process and primary metabolic process. Also, 32 proteins (60.37%) were involved in the catalytic activity process, which was the function that contained the most proteins of all the GO (Gene Ontology) annotations.

**Table 1:** All 53 proteins of *Ts*-ML-EVs recognized by 25 dpi swine serum were identified by LC-MS/MS

Accession	Protein names	Gene names	MW (kDa)	Abundances
A0A0V1BD93	Transmembrane protease serine 9	Prss30	126.2	9.22E+09
A0A0V1APR5	Snake venom 5'-nucleotidase	T01_175	61.9	4.36E+08
A0A0V1BSV1	ADP-ribose pyrophosphatase, mitochondrial	Nudt9	58.9	2.33E+08
A0A0V1C075	Deoxyribonuclease-2-alpha (fragment)	Dnase2	142.9	3.02E+09
A0A0V1AKL7	Metallophos domain-containing protein (fragment)	T01_11872	28.5	6.30E+06
A0A0V1ASF3	ADP-ribose pyrophosphatase, mitochondrial	Nudt9	54.5	1.63E+08
E5RYV9	Transmembrane protease serine 9	Tmprss9	71.6	3.37E+07
A1BQX7	Multi cystatin-like domain protein	mcd-1	45.9	7.45E+07
A0A0V1B2X1	Uncharacterized protein	T01_5772	50.7	6.47E+07
A0A0V1AS64	ADP-ribose pyrophosphatase, mitochondrial	Nudt9	57.2	4.31E+06
A0A0V1AVV5	Peptidase inhibitor R3HDML (fragment)	R3HDML	82	4.09E+07
A0A0V1BXV2	Mothers against decapentaplegic homolog	Mad	284.6	1.04E+08
A0A0V1ASF1	53 kDa excretory/secretory antigen (predicted)	T01_13886	41.2	4.52E+07
A0A0V1BH58	Ubiquitin–protein ligase (predicted)	T01_8770	37.2	9.59E+06
A0A0V1AX39	Fructose-bisphosphate aldolase	T01_11246	43.6	4.08E+06
A0A0V1BT87	2-Phospho-D-glycerate hydro-lyase (Fragment)	enol-1	50.3	2.98E+06
A0A0V1BNJ5	Malate dehydrogenase (fragment)	MDH2	109.1	1.72E+06
A0A0V1C1L0	Uncharacterized protein	T01_11591	36.2	2.06E+07
E5S9D6	Apple domain-containing protein	T01_1244	49.9	1.24E+07
A0A0V1B1S0	Putative nudix hydrolase 6	ndx-6	51	1.31E+07
A0A0V1AYM6	Peptidase S1 domain-containing protein	T01_6497	93.1	7.15E+06
E5RYD6	Peptidase S1 domain-containing protein (predicted)	T01_2202	31.2	1.98E+07
A0A0V1BT34	53 kDa excretory/secretory antigen (predicted)	T01_4139	34	8.55E+06
A0A0V1AZ55	Phosphoenolpyruvate carboxykinase (GTP)	PEPCK	83	5.03E+06
A0A0V1BN13	Sodium/potassium-transporting ATPase subunit beta-1	ATP1B1	303.9	3.92E+06
A0A0V1BXN8	Malic enzyme	Me3	137.3	1.50E+06
A0A0V1BCK0	Mannose-6-phosphate isomerase	T01_7294	484	1.75E+07
A0A0V1BWP3	Cysteinylglycine-S-conjugate dipeptidase	lap3	57.2	1.20E+07
A0A0V1B1P7	DDE_Tnp_1_7 domain-containing protein	T01_8160	51.1	1.93E+06
A0A0V0YZ53	Transmembrane protease serine 2 (fragment)	Tmprss2	10	
A0A0V1BW27	Glioma pathogenesis-related protein 1	T01_3621	52.7	7.07E+05
A0A0V1ARS7	ADP-ribose pyrophosphatase, mitochondrial	NUDT9	26.8	5.76E+06
A0A0V1AVL6	Tissue-type plasminogen activator	PLAT	44.2	
A0A0V1BP23	Cleavage and polyadenylation specificity factor subunit 2	Cpsf2	125.5	2.89E+06
A0A0V1AUA7	Transmembrane protease serine 9	TMPRSS9	152.1	1.59E+06
A0A0V1BIS9	Serine protease 28	Prss28	34	5.41E+06
E5SLU1	Actin-5C	Act5C	41.8	5.24E+06
A0A0V1BW41	Glutamate dehydrogenase (NAD(P)(+))	GLUD2	115.4	
A0A0V1BIH7	SML-4	sml-4	15.7	1.35E+06
A0A0V1BU64	PAN domain protein (predicted)	T01_14583	107.5	
A0A0V1BUA3	BAR domain-containing protein (fragment)	T01_4307	47.1	1.08E+07
A0A0V1B2I0	Plasma kallikrein	Klkb1	35.2	8.31E+06
A0A0V1AU45	ATP synthase subunit alpha (fragment)	ATP5A1	61.9	3.63E+06
A0A0V1B0A9	Tetratricopeptide repeat protein 37	TTC37	192.9	4.44E+06
A0A0V1C272	Uncharacterized protein	T01_3299	12.7	5.70E+05
A0A0V1BM94	Uncharacterized protein	T01_10190	13	
A0A0V1B8T3	Chymotrypsinogen B (fragment)	CTRB1	33.6	1.14E+06
A0A0V1BVG9	Trans-2-enoyl-CoA reductase, mitochondrial (fragment)	MECR	40.4	6.80E+05
A0A0V1B1B9	3-Ketoacyl-CoA thiolase, mitochondrial (fragment)	ACAA2	78	6.81E+05
A0A0V1BBH0	Secreted from muscle stage larvae 1 (predicted)	T01_15448	32.2	
A0A0V1B416	Peregrin	BRPF1	132.4	
E5SUQ1	Enteropeptidase	Tmprss15	75.1	
A0A0V1BNM9	Non-specific serine/threonine protein kinase	ATR	298.4	5.22E+06

### Bioinformatic analysis of *Ts*-TTPA sequences

**Fig. 4:**
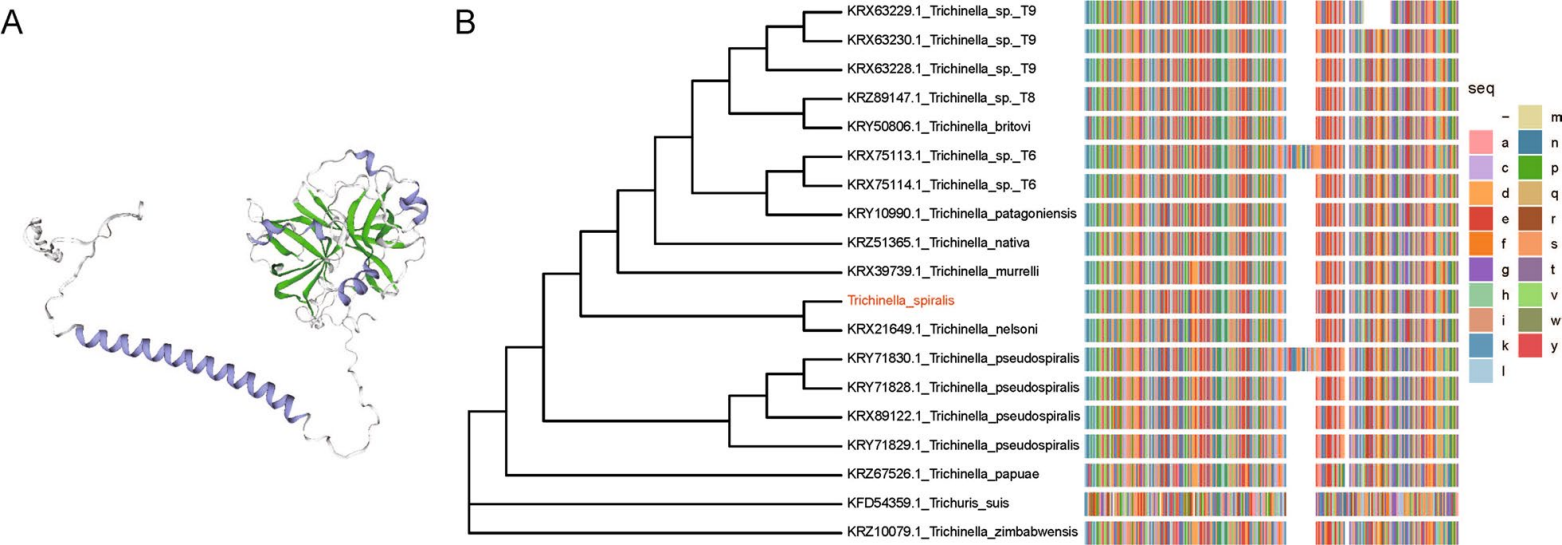
Bioinformatic analysis of *Ts*-TTPA. **A** Three-dimensional structural model of *Ts*-TTPA protein with secondary structure display. The alpha helix is covered in purple, the beta fold is covered in green, and the ring area is covered in gray. **B** Cladogram of analysis of *Ts*-TTPA. The maximum parsimony tree of tissue-type plasminogen activator protein in 12 species of *Trichinella spiralis* and *Trichuris suis* was drawn in MEGA. Protein sequence thumbnails of *Ts*-TTPA for each species are shown on the right. Different-colored squares represent different amino acids

Bioinformatics analysis revealed that the full-length cDNA sequence of the *Ts*-TTPA gene was 1152 bp and contained 384 amino acids. The predicted MW of *Ts*-TTPA was 44.19 kDa and the isoelectric point was 8.81. Amino acids 1–24 (ATGAATAATAGAAATAAAATAAAA) were predicted to be its signal peptide, and the Pfam database showed the presence of trypsin domains at 118–371 aa. In addition, the Swiss model was used to predict 3D structures, showing secondary structures including α-helix, β-folding and curling (Fig. 4a). In addition, we performed an evolutionary analysis of this protein and found that it has the highest homology with *Trichinella nelsoni* (Fig. 4b).

### Expression and purification of r*Ts*-TTPA

**Fig. 5:**
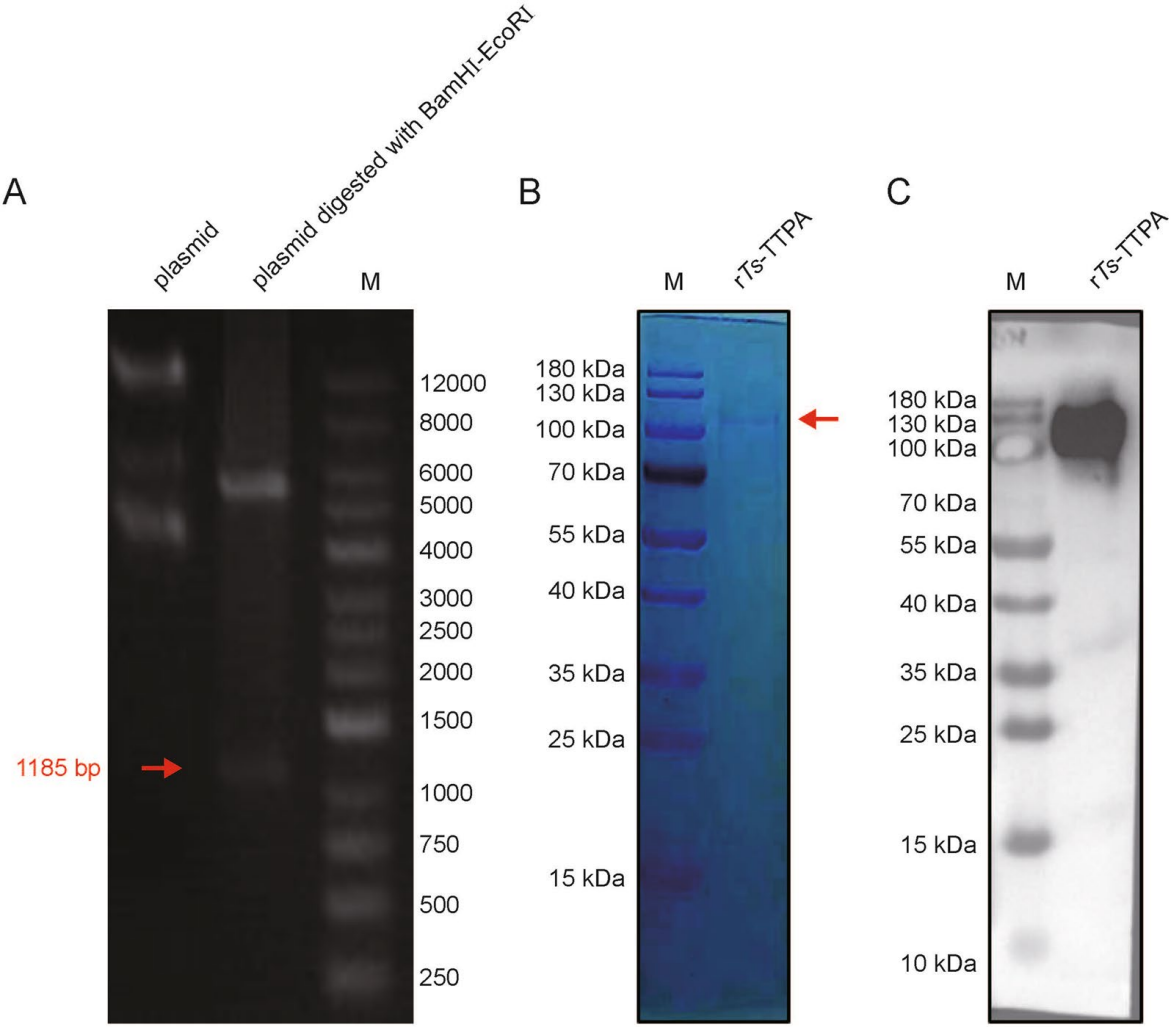
Expression and purification of r*Ts*-TTPA. **A** The constructed vector was identified by double restriction enzyme digestion. From right to left are DNA markers, pMAL-c5X-*Ts*-TTPA plasmid digested with BamHI/EcoRI and pMAL-c5X-*Ts*-TTPA plasmid. The red arrow shows the *Ts*-TTPA gene. **B** The purified *Ts*-TTPA was stained with Coomassie Bright Blue. The red arrow shows the *Ts*-TTPA protein. **C** The purified r*Ts*-TTPA was identified by monoclonal His-tag antibody

The expression vectors, PMAL-c5X-tissue-type plasminogen activator (UniProt: A0A0V1AVL6), were constructed successfully with His and MBP labels (Fig. 5a). The purified recombinant proteins were obtained by inducible expression and named r*Ts*-TTPA (Fig. 5b). The purified protein was identified by mouse-His tag antibody (Fig. 5c).

### Recognition of r*Ts*-TTPA by infected serum

**Fig. 6:**
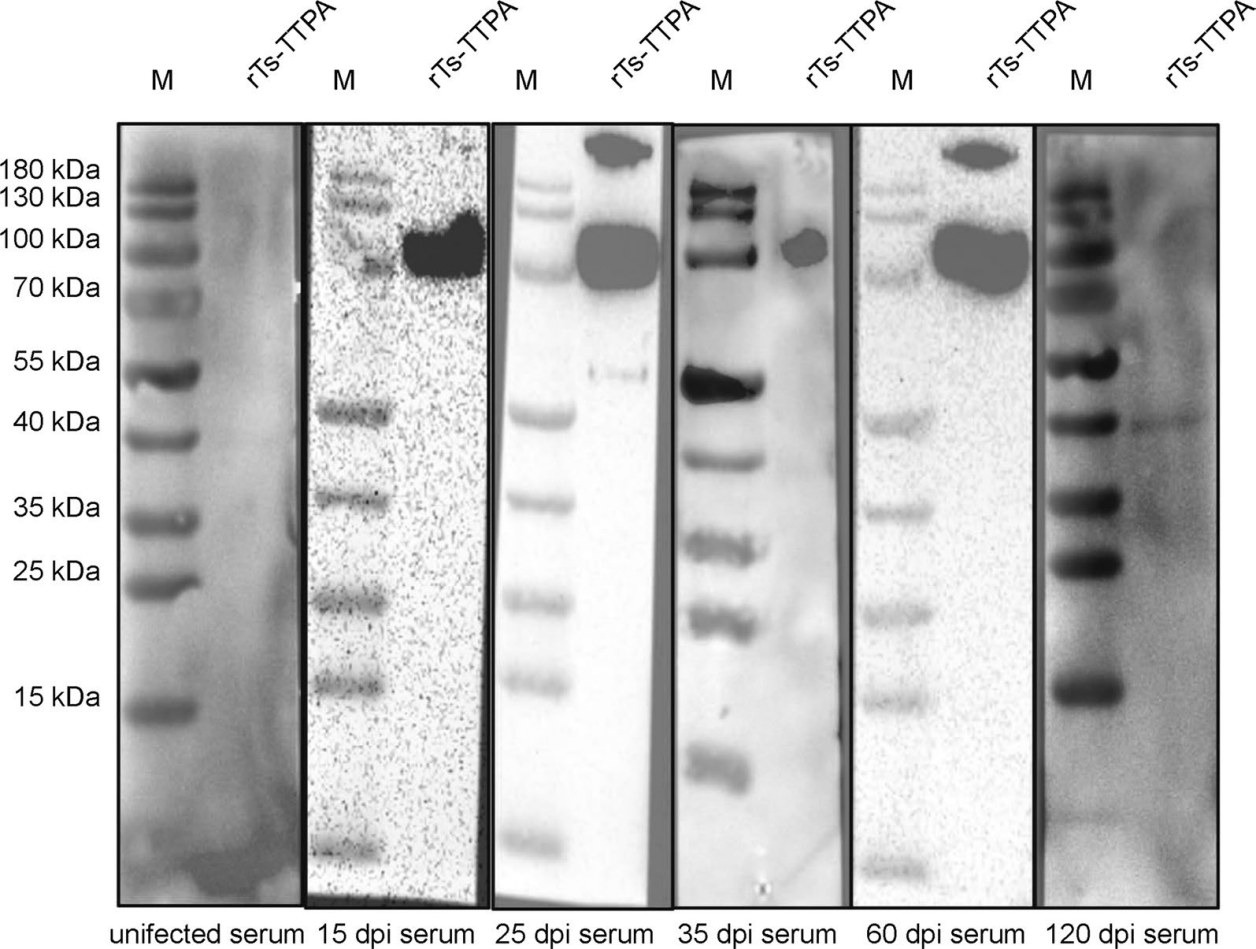
Identification of r*Ts*-TTPA reactivity. The r*Ts*-TTPA was recognized by infected swine serum at different times (15 dpi, 25 dpi, 35 dpi, 60 dpi, 120 dpi), and negative serum was used as a control

Subsequently, hybridization with swine serum post-infection on different days showed that there was no reaction with negative serum and serum at 120 dpi with 10,000 *T. spiralis*, while swine serum at 15, 25, 35 and 60 dpi with 10,000 *T. spiralis* could recognize this protein, with the earliest detection time being 15 dpi (Fig. 6).

### Dynamics of serum anti-*Trichinella* IgG in experimentally infected pigs

**Fig. 7:**
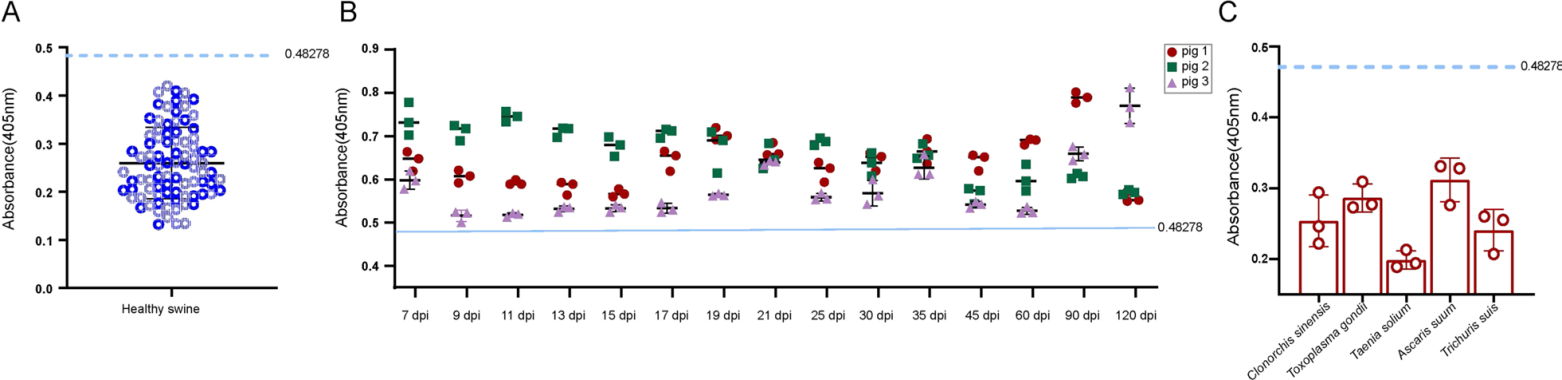
Establishment of r*Ts*-TTPA-ELISA for early stage detection. **A** Eighty-six negative pig serum samples (1:100) were used to calculate the cut-off, with line representing mean + 3 SD (blue). **B** Kinetics of serum anti-*Trichinella* IgG in pigs experimentally infected with 10,000 muscle larvae (*n* = 3). **C** Cross-reactions assay of serum infected with *Clonorchis sinensis*, *Toxoplasma gondii*, *Taenia suis*, *Ascaris suis* and *Trichuris suis* (*n* = 3)

Serum anti-*Trichinella* IgG levels of pigs at different time intervals were detected by ELISA using r*Ts*-TTPA. By testing 86 negative samples, we determined that the cut-off value was 0.48278 (Fig. 7a). The r*Ts*-TTPA-ELISA can detect the specific anti-*Trichinella* IgG at 13 dpi, with a positive rate of 100% in the detected samples (Fig. 7b). In addition, the results from the cross-reaction experiments showed that the detection value was lower than the cut-off line, which indicated that the r*Ts*-TTPA-ELISA had no cross-reactions with other common swine parasites and had good specificity (Fig. 7c).

## Discussion

*Trichinella spiralis* is an important zoonotic disease, and its detection has always been a hot topic in research. Although molecular detections have been widely reported, serological detection is the most widely used detection method for trichinellosis and is recommended by the World Health Organization (WHO) and the International Commission on Trichinellosis (ICT) [24]. Previous studies have reported the detection of trichinellosis using circulating antigens such as crude extract protein and excretory-secretory products as detected molecules, but there is an obvious detection window period limited to within 2 or 3 weeks of infection [25]. The ML-ES antigen-ELISA has been used consistently in our laboratory to detect IgG in serum from pigs infected with *T. spiralis*, and IgG detection is not complete until 19 dpi [5]. Therefore, improving detected antigens has clinical value, and increasingly studies are focusing on screening antigens for early stage detection [4].

In recent years, research on parasite-derived extracellular vesicles has attracted much attention [26]. Parasite-derived extracellular vesicles can be used as signaling molecules to participate in parasite-host interactions, maintain the parasitism of parasites and cause host diseases. Extracellular vesicles also participate in host defense responses through antigen presentation [27–29]. Proteomic and transcriptomic analysis revealed that parasite-derived extracellular vesicles contain a large number of proteins and non-coding RNAs, which are involved in parasite reproduction, survival and host immune regulation, and also elucidated a novel parasite-host communication mode mediated by parasite-derived extracellular vesicles [30, 31]. In addition, compared with other sources, the extracellular vesicles collected in this study are enriched and may detect antigenic proteins that have not been previously reported, providing new candidate molecules for the diagnosis of *T. spiralis* infection.

We screened 53 proteins from *Ts*-ML-EVs by LC-MS/MS, and besides being involved in some basic metabolic pathways, they are also widely involved in the catalytic and hydrolase activity of *T. spiralis*, which have been reported to play important roles in the growth, development and invasion of *T. spiralis* [32, 33]. In addition, some proteins in *Ts-*ML-EVs have also been reported to be useful for early stage detection and candidate vaccines. Furthermore, we conducted extensive screening of unreported proteins to obtain new detected antigens or vaccine candidates.

The candidate molecule *Ts*-TTPA identified here has the trypsin domain, which belongs to the peptidase family S1, in which serine serves as the nucleophilic amino acid at the active site. It is the largest protease family, including catalytic active and non-catalytic active enzymes, known as "serine protease" and "serine protease homologs," respectively [34]. Some proteins with serine protease activity isolated from *T. spiralis* have been reported to be involved in parasite-host interactions, including immune regulation and the invasion of intestinal epithelium [35–37]. In addition, these antigenic proteins have been reported as candidate molecules for the diagnosis of antigens. Sun et al. used the recombinant expression of *Ts*-SP as an antigen to detect the serum of mice infected with 300 muscle larvae and found that they could detect the infected serum at 12 dpi at the earliest [6]. The *Ts*-TTPA we reported also had the same effect, and it was able to detect infected serum as early as 7 dpi. Three sera infected with 10,000 *T. spiralis* were fully detected at 13 dpi. The identification of these detected antigens partially supplemented the window period for the detection of IgG and provided a new reference for the early stage detection of *T. spiralis*. However, the sample size of this experiment is not large, which carries certain limitations, and relevant research and detection need to be further carried out. In addition, this *Ts*-TTPA can be expressed in large quantities and is easier to obtain than excretory-secretory product antigens. Due to the trypsin activity of this protein, future functional experiments and host interaction studies should be done.

Previous studies have also reported the protective efficacy of *Ts*-ML-EVs as a vaccine, but which molecules were not specified [15], although 53-kDa excretory/secretory antigens and a deoxyribonuclease-2-alpha antigen have been reported to have protective potential [38, 39]. In addition, whether some other proteins, including our candidate proteins, also play a role in the protective immunity related to *Ts*-ML-EVs needs to be further investigated.

## Conclusions

The extracellular vesicles of *T. spiralis* muscle larvae successfully isolated in this study have specific markers of extracellular vesicles. The protein binds in extracellular vesicles of *T. spiralis* identified by 25 dpi serum; especially the *Ts*-TTPA protein with trypsin domain can be the fastest to identify swine serum at 13 dpi and is a candidate antigen for early stage detection of trichinellosis.

## Data Availability

All data in this study are available in the data presented and additional files.

## References

[1] Bai X, Hu X, Liu X, Tang B, Liu M (2017). Current research of trichinellosis in China. Front Microbiol.

[2] Wang N, Bai X, Tang B, Yang Y, Wang X, Zhu H (2020). Primary characterization of the immune response in pigs infected with *Trichinella spiralis*. Vet Res.

[3] Brodaczewska K, Wolaniuk N, Lewandowska K, Donskow-Lysoniewska K, Doligalska M (2017). Biodegradable chitosan decreases the immune response to *Trichinella spiralis* in mice. Molecules.

[4] Sun GG, Wang ZQ, Liu CY, Jiang P, Liu RD, Wen H (2015). Early serodiagnosis of trichinellosis by ELISA using excretory-secretory antigens of *Trichinella spiralis* adult worms. Parasit Vectors.

[5] Wang N, Bai X, Ding J, Lin J, Zhu H, Luo X (2021). Trichinella infectivity and antibody response in experimentally infected pigs. Vet Parasitol.

[6] Sun GG, Song YY, Jiang P, Ren HN, Yan SW, Han Y (2018). Characterization of a *Trichinella spiralis* putative serine protease. Study of its potential as sero-diagnostic tool. PLoS Neglect Trop Dis.

[7] Wang ZQ, Liu RD, Sun GG, Song YY, Jiang P, Zhang X (2017). Proteomic analysis of *Trichinella spiralis* adult worm excretory-secretory proteins recognized by sera of patients with early trichinellosis. Front Microbiol.

[8] Gao H, Tang B, Bai X, Wang L, Wu X, Shi H (2018). Characterization of an antigenic serine protease in the *Trichinella spiralis* adult. Exp Parasitol.

[9] Théry C, Witwer KW, Aikawa E, Alcaraz MJ, Anderson JD, Andriantsitohaina R (2018). Minimal information for studies of extracellular vesicles 2018 (MISEV2018): a position statement of the International Society for Extracellular Vesicles and update of the MISEV2014 guidelines. J Extracell Vesicles.

[10] Wang Y, Liu J, Ma J, Sun T, Zhou Q, Wang W (2019). Exosomal circRNAs: biogenesis, effect and application in human diseases. Mol Cancer.

[11] Tzelos T, Matthews JB, Buck AH, Simbari F, Frew D, Inglis NF (2016). A preliminary proteomic characterisation of extracellular vesicles released by the ovine parasitic nematode. Teladorsagia circumcincta Vet Parasitol.

[12] Samoil V, Dagenais M, Ganapathy V, Aldridge J, Glebov A, Jardim A (2018). Vesicle-based secretion in schistosomes: Analysis of protein and microRNA (miRNA) content of exosome-like vesicles derived from *Schistosoma mansoni*. Sci Rep.

[13] Rooney J, Northcote HM, Williams TL, Cortés A, Cantacessi C, Morphew RM (2022). Parasitic helminths and the host microbiome—A missing 'extracellular vesicle-sized' link?. Trends Parasitol.

[14] Kifle DW, Pearson MS, Becker L, Pickering D, Loukas A, Sotillo J (2020). Proteomic analysis of two populations of *Schistosoma mansoni*-derived extracellular vesicles: 15k pellet and 120k pellet vesicles. Mol Biochem Parasitol.

[15] Gao X, Yang Y, Liu X, Xu F, Wang Y, Liu L (2022). Extracellular vesicles from *Trichinella spiralis*: Proteomic analysis and protective immunity. PLoS Negl Trop Dis.

[16] Liu Y, Xu N, Li Y, Tang B, Yang H, Gao W (2021). Recombinant cystatin-like protein-based competition ELISA for *Trichinella spiralis* antibody test in multihost sera. PLoS Negl Trop Dis.

[17] Li F, Cui J, Wang ZQ, Jiang P (2010). Sensitivity and optimization of artificial digestion in the inspection of meat for *Trichinella spiralis*. Foodborne Pathog Dis.

[18] Protein Structure Prediction Server (PSIPRED). http://bioinf.cs.ucl.ac.uk/psipred Accessed 28 Apr 2023.

[19] Pfam. http://pfam-legacy.xfam.org/ Accessed 28 Apr 2023.

[20] SWISS-MODEL. https://swissmodel.expasy.org/ Accessed 28 Apr 2023.

[21] ProtParam. https://www.expasy.org/resources/protparam Accessed 28 Apr 2023.

[22] Gene Ontology Resource. http://geneontology.org/ Accessed 20 Mar 2023.

[23] Kumar S, Stecher G, Li M, Knyaz C, Tamura K (2018). MEGA X: molecular evolutionary genetics analysis across computing platforms. Mol Biol Evol.

[24] Bruschi F, Gómez-Morales MA, Hill DE (2019). International Commission on Trichinellosis: Recommendations on the use of serological tests for the detection of *Trichinella* infection in animals and humans. Food Waterborne Parasitol.

[25] Wang ZQ, Shi YL, Liu RD, Jiang P, Guan YY, Chen YD (2017). New insights on serodiagnosis of trichinellosis during window period: early diagnostic antigens from *Trichinella spiralis* intestinal worms. Infect Dis Poverty.

[26] Ofir-Birin Y, Regev-Rudzki N (2019). Extracellular vesicles in parasite survival. Science.

[27] Greening DW, Xu R, Ale A, Hagemeyer CE, Chen W (2023). Extracellular vesicles as next generation immunotherapeutics. Semin Cancer Biol.

[28] Marcilla A, Martin-Jaular L, Trelis M, de Menezes-Neto A, Osuna A, Bernal D (2014). Extracellular vesicles in parasitic diseases. J Extracell Vesicles.

[29] Hansen EP, Fromm B, Andersen SD, Marcilla A, Andersen KL, Borup A (2019). Exploration of extracellular vesicles from *Ascaris suum* provides evidence of parasite-host cross talk. J Extracell Vesicles.

[30] Nowacki FC, Swain MT, Klychnikov OI, Niazi U, Ivens A, Quintana JF (2015). Protein and small non-coding RNA-enriched extracellular vesicles are released by the pathogenic blood fluke *Schistosoma mansoni*. J Extracell Vesicles.

[31] Wu Z, Wang L, Li J, Wang L, Wu Z, Sun X (2018). Extracellular vesicle-mediated communication within host–parasite interactions. Front Immunol.

[32] Song YY, Zhang Y, Ren HN, Sun GG, Qi X, Yang F (2018). Characterization of a serine protease inhibitor from *Trichinella spiralis* and its participation in larval invasion of host's intestinal epithelial cells. Parasit Vectors.

[33] Wang J, Jin X, Li C, Chen X, Li Y, Liu M (2023). In vitro knockdown of TsDNase II-7 suppresses *Trichinella spiralis* invasion into the host's intestinal epithelial cells. PLoS Negl Trop Dis.

[34] Page MJ, Di Cera E (2008). Serine peptidases: classification, structure and function. Cell Mol Life Sci.

[35] Xue Y, Zhang B, Wang N, Huang HB, Quan Y, Lu HN (2022). Oral vaccination of mice with *Trichinella spiralis* putative serine protease and murine interleukin-4 DNA delivered by invasive *Lactiplantibacillus plantarum* elicits protective immunity. Front Microbiol.

[36] Long SR, Liu RD, Kumar DV, Wang ZQ, Su CW (2021). Immune protection of a helminth protein in the DSS-induced colitis model in mice. Front Immunol.

[37] Li C, Bai X, Liu X, Zhang Y, Liu L, Zhang L (2021). Disruption of epithelial barrier of caco-2 cell monolayers by excretory secretory products of *Trichinella spiralis* might be related to serine protease. Front Microbiol.

[38] Xu D, Tang B, Yang Y, Cai X, Jia W, Luo X (2021). Vaccination with a DNase II recombinant protein against *Trichinella spiralis* infection in pigs. Vet Parasitol.

[39] Xu D, Bai X, Xu J, Wang X, Dong Z, Shi W (2021). The immune protection induced by a serine protease from the *Trichinella spiralis* adult against *Trichinella spiralis* infection in pigs. PLoS Negl Trop Dis.

